# Sexual and reproductive health of adolescents and young people in the Gambia: a systematic review

**DOI:** 10.11604/pamj.2021.40.221.25774

**Published:** 2021-12-13

**Authors:** Mat Lowe, Phebian Ina Grant Sagnia, Olusegun Awolaran, Yves Armand Médessi Mongbo

**Affiliations:** 1 Society for the Study of Women’s Health (SSWH), Old Yundum, Coastal Road, The Gambia,; 2 Directorate of Health Research, Ministry of Health, Old Yundum, Coastal Road, The Gambia,; 3 West African Health Organization (WAHO), Bobo-Dioulasso, Burkina Faso

**Keywords:** Gambia, adolescents, young people, sexual health, reproductive health, systematic review

## Abstract

**Introduction:**

in the Gambia, adolescents and young people make up 32% of the national population. Observations indicate that this population group lack access to quality information and services on sexual and reproductive health. This systematic review explored some sexual and reproductive health indicators relevant to adolescents and young people in the Gambia.

**Methods:**

published studies on some sexual and reproductive health indicators were searched in PubMed, Google Scholar and African Journals Online. Moreover, national reference documents containing relevant in-country data on adolescents and young people's sexual and reproductive health were also collected and reviewed. Search terms for published studies focused on modern contraceptives, sexually transmitted infections (STIs) prevalence rates, availability and accessibility to sexual and reproductive health (SRH) services and adolescents and young people’s satisfaction with SRH services among adolescents and young people (aged 10-24 years).

**Results:**

the review showed that contraceptive prevalence rates among adolescents and young people in Gambia ranged from 7% to 9%. Reasons for low contraceptive prevalence among adolescents and young people included limited knowledge and access to sexual and reproductive health information and services, provider attitudes, stigma, shame, lack of money, cultural and religious misconceptions associated with contraception. Overall, the review found limited information on STI prevalence among adolescents and young people, with a single published study reporting a prevalence rate of 8.4%. In addition, inadequate counseling, complaints related to physical environment as well as the process of providing sexual and reproductive health services and information were significant factors associated with satisfaction with SRH services among adolescents and young people.

**Conclusion:**

this review provides important baseline data that may be useful for policymakers and program managers to improve adolescents and young people's sexual and reproductive health in Gambia.

## Introduction

Concerns about adolescents and young people’s sexual and reproductive health (AYSRH) have grown over the past years because of the unprecedented increase in the number of young people, rates of sexual and reproductive health infections, early sexual debut, teenage and unwanted pregnancies, and gender-based violence, including female genital mutilation/cutting. Teenage pregnancies outside of marriage among adolescent girls, in particular, are a significant cause for concern in The Gambia, where 18% of adolescent girls, age 15 to 19 are already mothers or pregnant with their first child [1]. In addition, teen pregnancies constitute a significant cause of unsafe abortions, contributing to 13% of Gambia´s maternal deaths [2]. Even though there is paucity of data on the prevalence of adolescent pregnancies and abortions, in 2011 alone, there were 19 reported cases of abandoned babies [3]. These figures increased to 20 in 2012 and 25 in 2013 [4].

In addition to the complications which might lead to death, other consequences for an unmarried adolescent and young person with pregnancy include expulsion from home, dishonor for her family and herself, and child neglect and abuse [5]. The underlying causes of unplanned pregnancies among adolescents and young people are many. These include lack of timely and appropriate information from parents on sexual and reproductive health; denial of adolescents and young people's access to sexual and reproductive health information and services [6]. Misinformation about sex and the lack of youth-friendly sexual and reproductive health services in the Gambia also pose challenges to young girls' sexual and reproductive health [6]. These issues have all contributed to the high prevalence of HIV/AIDS in the country, which is higher among females, 2.1%, compared to males, 1.7% [7]. Knowledge of HIV transmission is also limited in the Gambia, with only 27 percent of women and 36 percent of men age 15-49 having comprehensive knowledge about AIDS.

Although there are policy frameworks regarding sexuality education to empower adolescents and young people with knowledge and practical skills to make safe and informed choices and exercise their rights, there is limited evidence on the effectiveness of these policy frameworks. Close observations indicate that adolescents and young people (aged 10-24) who constitute 32% of the nation’s population, lack quality information and services on sexual and reproductive health. This systematic review explores some sexual and reproductive health indicators of adolescents and young people in the Gambia. The review was part of a more extensive baseline study of sexual and reproductive health of adolescents and young people in the Gambia, initiated by the West African Health Organization (WAHO).

## Methods

**Reporting**: the Preferred Reporting Items for Systematic Review and Meta-analyses (PRISMA) guideline [8] was used to report this study.

**Search strategies and information sources**: an extensive electronic literature search to identify terms related to sexual and reproductive health of adolescents and young people in the Gambia was conducted. Search terms focused mainly on modern contraceptive and STIs prevalence rates, availability and accessibility of sexual and reproductive health (SRH) services, and satisfaction with SRH services among adolescents and young people (aged 10-24 years). Electronic literature searches were conducted in accessible databases including PubMed, Google Scholar and African Journals Online. These databases were searched without restriction to date. Search terms are described in Table 1.

**Table 1:** search terms

Databases
PubMed, Google Scholar, African Journals Online
**PubMed search terms**
“availability and access of reproductive health services” OR ”Gambia “contraceptive prevalence” OR “Gambia” “teenage pregnancy” OR “Gambia”, “STIs prevalence Gambia” “Sexual health”
**Google Scholar search terms**
“adolescent health Gambia” “sexual health Gambia” “early pregnancy Gambia” “STIs prevalence Gambia”
**African Journals Online search terms**
“Reproductive health”, “sexual health Gambia” “contraceptive Gambia”

**Figure 1:**
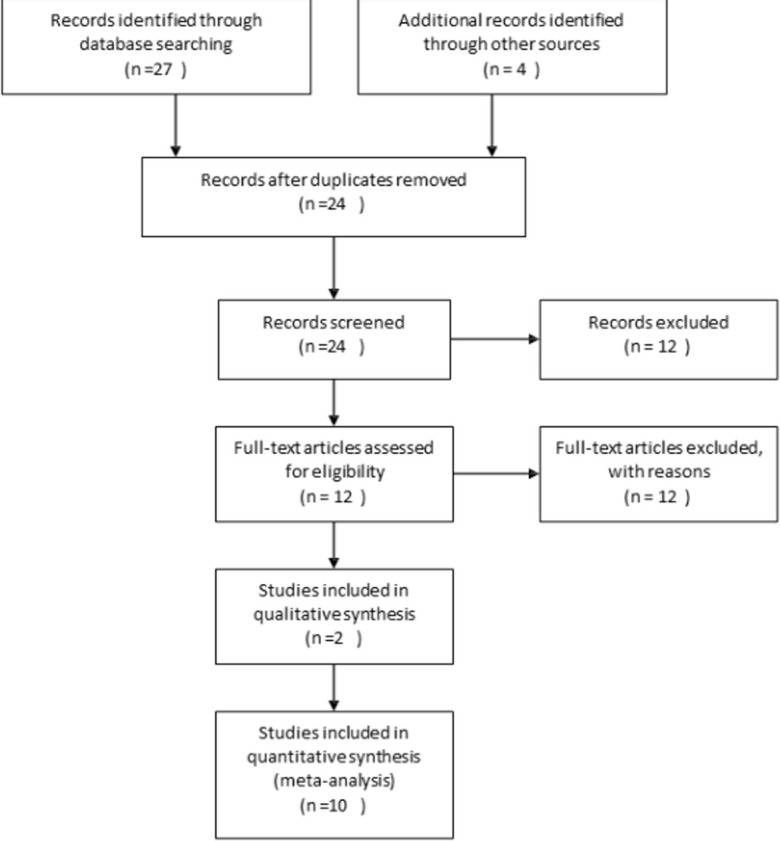
PRISMA 2009 Flow diagram

**Study selection and eligible criteria**: the studies included in this review were peer-reviewed published studies in English and national reference documents, including project, programs and evaluations reports, without restriction to date. Published studies and national reference documents that provided no information on sexual and reproductive health of adolescents and young people were excluded. The study population of interest included adolescents and young people. For adolescents and young people, the study applied the official definitions of the UN. The UN defines adolescents as individuals being 10-19 years old and young people as those persons between the ages of 15 and 24 years. A total of 27 published, peer-reviewed articles were identified through the keyword search that was conducted on April 15, 2020. A title and abstract screening to determine final inclusion of studies in the review was then conducted and this resulted in the exclusion of 12 articles that were deemed irrelevant and or lack data on sexual and reproductive health of 12 articles that were deemed irrelevant and or lack data on sexual and reproductive health of adolescents and young people (Figure 1). Beside the studies that were obtained from the electronic literature search, national reference documents that contained relevant in-country data on sexual and reproductive health of adolescents and young people were also collected from relevant government and non-governmental agencies (Table 2). A total of 7 national reference documents were collected. Of these, 3 were included in this review.

**Table 2:** list of government and non-government agencies for in-country data

Name	Classification/ type
Gambia Bureau of Statistics (GBoS)	Government
Ministry of Health (Reproductive, Maternal, Neonatal, Child and Adolescent Health Unit)	Government
National Youth Council (NYC)	Government
National AIDS Secretariat (NAS)	Government
UNFPA	Non-government/ UN agency
Gambia Family Planning Association (GFPA)	Non-government
Society for the Study of Women´s Health (SSWH)	Non-government

**Data extraction and analysis**: one of the authors (OA) designed a data extraction form. The other authors (ML, PIGS, and YAMM) independently checked the data extraction form for accuracy. The data extraction was then done by (ML and PIGS) to extract the following information from each included study; study ID, author, year of publication, data source, study area, study design, population and setting, the age range of study participants, mean age of participants, sample size, and outcome measures including contraceptive and STIs prevalence rates, availability and accessibility of sexual and reproductive health services, level of satisfaction with sexual and reproductive health services. Since this was a rapid systematic review, the authors did not assess the risk of bias for the studies included in the review. In addition, due to the inclusion of qualitative and quantitative studies, conducting a meta-analysis of the data was not appropriate. Instead, the authors chose a descriptive narrative synthesis as the most suitable data synthesis method for this review.

## Results

**Results of the search and characteristics of the studies included**: the literature searches from all sources yielded a total of twelve (12) published studies and three national reference documents that met the inclusion criteria in this review. The published studies consisted of two (2) qualitative and ten (10) quantitative studies as well as the three national reference documents, which were reports of Multiple Indicator Cluster Survey (MICS), Demographic and Health Survey (DHS) and a rapid assessment study (Table 3, Table 3 (suite)).

**Table 3:** characteristics of studies included in the review

N	Author	Year	Study Population	Age range	Study design	Main research objective/
1	Ba DM *et al*. [9]	2019	Women of reproductive age	15-49	Population-based study	To measure the prevalence of contraceptive use among married adolescents in 17 sub-Saharan Africa countries and identify factors associated with contraceptive use in these countries
2	Kane TT [10]	1993	Young men and women	14-24	Two-stage probability sample survey	To explore sexual activity, family life education, and contraceptive practice among young adults in Banjul, The Gambia.
3	Wilkins HA [11]	1989		15	Population-based cross sectional study	To determine knowledge of AIDS, use of condoms and results of counselling subjects with asymptomatic HIV2 infection in The Gambia
4	Anyanwu M *et al*. [12]	2017	Women of reproductive age	15-49	A facility based prospective cross-sectional study	To determine the uptake of long-acting reversible contraceptive in The Gambia.
5	Netw Res Triangle Park NC [13]	1988	Single and married women	14-24	Not mentioned	To develop, evaluate, and improve programs for young people by providing information about fertility, contraceptive use, and reproductive health problems among young people
6	Kane TT *et al*. [14]	1990	Male and female youth	14-24	Knowledge, attitude, and practice (KAP) study	To assess knowledge, attitude, and practice towards adolescent pregnancy and contraception in greater Banjul

**Table 3(suite):** characteristics of studies included in the review

N	Author	Year	Study Population	Age range	Study design	Main research objective/
7	Centre Français sur la Population de le Développement CEPED [15]	1997	Adolescents	15-20	Not mentioned	To study adolescent sexuality in the Sahel
8	Miles K *et al*. [16]	2001	Men and women	15-25	Not mentioned	To explore the sexual health treatment-seeking behaviors of young people
9	Niki Cotten *et al*. [17]	1992	New contraceptive users	Not mentioned	Prospective study	To assess the extent of and reasons for contraceptive discontinuation within the first eight months of acceptance
10	Jammeh *et al*. [18]	2014	Married men and women	Not mentioned	Community-based cross sectional study	To explore married couples' family planning knowledge, attitudes, and practices in rural and urban Gambia and to analyze what factors may affect such knowledge, attitudes and practices.
11	Jallow IK *et al*. [20]	2012	Women of reproductive age	15-49	Descriptive cross-sectional study	To assess women's preferences and perception of antenatal healthcare services in public and private healthcare facilities.
12	Isatou Dampha *et al*. [21]	2018	Women	Not mentioned	Phenomenological qualitative study design	To describe the views, opinions, life experiences and perspectives of the study participants concerning the use of levonorgestrel implants.

**Contraceptive prevalence**: three studies reported information on contraceptive prevalence. The reported contraceptive prevalence rates among married and unmarried adolescents and young people aged 10-24 years ranged from 7%-8% in the three studies [9-11] and 9% in a national survey [12].

**Availability and accessibility of contraceptives**: lack of access and limited knowledge of reproduction and reproductive health were reported in two studies as main reasons for non-use of modern contraceptives among adolescents and young people aged 10-24 years [13,14]. Other reasons for non-use of contraceptives and other needed reproductive health services by adolescents and young people are the disapproving attitude of health workers [15], stigma, shame and lack of money [16]. The low uptake of contraception by young women and girls was also attributed to cultural and religious barriers [17] and common misconception which associate contraception with promiscuity in a study [18].

**Prevalence of sexually transmitted infection**: there is limited information on the prevalence of STI among adolescents and young people, with only a single study reporting a prevalence rate of 8.4% among married adolescent and young girls aged 15-24 [19].

**Satisfaction with sexual and reproductive health services**: inadequate counselling and complaints relating to physical environment and process of providing sexual and reproductive health services and information were reported as major factors associated with level of adolescent and young women´s satisfaction with the quality of sexual and reproductive health services they received [20].

## Discussion

This study was a systematic literature review of published studies and national reference documents on sexual and reproductive health of adolescents and young people in the Gambia. The studies and national reference documents included in the review show poor sexual and reproductive health among adolescents and young people. As a result, the uptake of modern contraceptive methods is low and contributes to the high pregnancy rates among adolescent girls. The studies also revealed that the low uptake of contraception by adolescents and young people can be attributed to cultural and religious barriers including misconceptions which associate contraception with promiscuity; shame and the disapproving attitude of health workers. These all contribute to the reasons why young people do not access health services, even if they had STI symptoms. Furthermore, there is limited information on the prevalence of STI among adolescents and young people, with just one published study reporting an STI prevalence of 8.4% [19]. This suggests the need to conduct more research to assess the prevalence of STIs among adolescents and young people in the Gambia. The review also explored levels of satisfaction with the quality of sexual and reproductive health services among adolescents and young people and found inadequate counselling, complaints relating to physical environment of the counselling area and process of providing sexual and reproductive health services and information as reasons for discontinuation and non-use of sexual and reproductive health commodities and services [21].

**Study limitations**: only studies which included results on adolescent and young people (10–24 years old) were included in this review. The fact that the studies included in the review were also limited to studies written in English is another limitation. Including ages beyond 10-24 years and studies written in other languages would have provided valuable information on sexual and reproductive health of adolescents and young people. In addition, although PubMed, Google Scholar and African Journals Online are among the most used search databases, the review might have missed some relevant studies included in other databases [22]. Some peer-reviewed studies which could not be accessed were also not included. The quality of the included studies was also not described in this review since the risk of bias for included studies was not assessed. Despite these limitations, the review has important implications for policy and research.

## Conclusion

The findings of this systematic review provide essential data on some sexual and reproductive health indicators of adolescents and young people in the Gambia. The study has demonstrated the gaps in evidence on adolescents and young people's sexual and reproductive health in the Gambia. It stressed the need to conduct more studies targeting adolescents and young people because majority of studies conducted on SRH in the Gambia are related to women of reproductive age, which may conceal the current state and needs of the adolescents with those of older women who are still within the reproductive age [22].

### What is known about this topic

- Adolescents and young people are often vulnerable to sexual and reproductive health problems;
- Due to their limited knowledge and access to sexual and reproductive health services, adolescents and young people face sexual and reproductive health problems, such as STIs and unplanned and unwanted pregnancies.

### What this study adds

- Adolescents and young people in the Gambia have limited access to sexual and reproductive health services;
- Healthcare providers, stigma, shame, lack of money, and cultural and religious misconceptions, limit adolescents and young people's access to sexual and reproductive health in the Gambia.
